# Effect of irrigant activation systems and dentine ageing on sealer penetration into dentinal tubules

**DOI:** 10.1590/1807-3107bor-2026.vol40.077

**Published:** 2026-10-16

**Authors:** Noelia SANTAMARIA, Jaime BASCONES, Carlos GALLEGO-GARCIA, Lucia GANCEDO-CARAVIA

**Affiliations:** (a) Complutense University, School of Dentistry, Department of Conservative Dentistry and Prosthetics, Madrid, Spain.; (b) Centro de Biología Molecular Severo Ochoa - CSIC-UAM, Advanced Light Microscopy Facility, Madrid, Spain.

**Keywords:** Microscopy, Confocal, Dentin, Root Canal Irrigants, Silicate Cement

## Abstract

This study aimed to assess the effect of dentine ageing and irrigant activation (ultrasonic activation (UA) and SmartLite Pro EndoActivator (SLP-EA)) on the penetration of endodontic sealer into dentinal tubules in the apical third. Eighty single-rooted teeth extracted from donors aged 30–40 years (n = 40) and 60–70 years (n = 40) were prepared using the ProTaper Gold system and randomly assigned to two irrigation protocols (UA or SLP-EA). Root canals were obturated with BC Universal Sealer labelled with sodium fluorescein using a single-cone technique. Roots were sectioned 3 mm from the apex and analysed using confocal laser scanning microscopy. Sealer penetration was quantified by total penetration area (PA), maximum penetration depth (MaxD), mean penetration depth (S-MeanD), and percentage of perimeter penetration at different depths. Data were analysed using 2-way ANOVA test (α = 0.05). No significant differences were observed between UA and SLP-EA, whereas donor age significantly affected sealer penetration. Younger teeth exhibited greater penetration than older teeth, with higher MaxD (1,458 µm vs. 698 µm), S-MeanD (738 µm vs. 183 µm), and perimeter percentages (p < 0.001). Within the limitations of this study, it may be concluded that dentine ageing affects the penetration of endodontic sealer into dentinal tubules in the apical third, regardless of the technique used to activate the irrigant. This finding should be considered when interpreting the results of experimental studies and emphasises the need to standardise donor age in tooth selection for *in vitro* studies.

## Introduction

The ultimate goal of endodontic therapy is to prevent or resolve apical periodontitis by eliminating bacteria and tissue remnants from the root canal system and achieving three-dimensional obturation.^1^ For endodontic treatment to be successful, proper chemical-mechanical preparation of the root canal must be performed. Despite advances in rotary instrumentation, several studies have demonstrated that mechanical preparation alone cannot completely clean the root canal system, allowing tissue remnants, biofilms, and debris to persist,^2^ particularly in areas such as accessory canals and dentinal tubules.^3^ This highly complex anatomy is especially relevant in the apical third of the root canal system, containing up to 73.5% of accessory canals and ramifications.^4^

Tubule density and patency decrease significantly with age due to progressive sclerosis and peritubular dentine formation.^5,6^ These changes occur more rapidly in the apical region^7^, often leading to partial or complete tubule obliteration.^6^ Thus, infiltration of irrigants and sealers into the dentinal walls at the apical end of the root canal may be more restricted in older patients than in younger patients.

Irrigation is used to clean areas that mechanical instrumentation cannot reach.^8^ Irrigants can be activated using different techniques, such as ultrasonic activation (UA). Through acoustic microstreaming and cavitation effects, it optimises debridement, smear layer and bacterial elimination and promotes deeper irrigant penetration into dentinal tubules.^9,10^ However, its efficacy may be reduced when the oscillating tip contacts the canal walls.^11^ EndoActivator system (EA, Dentsply Sirona, Ballaigues, Switzerland), is a sonic agitation device with flexible polymer tips designed to promote active fluid movement inside the root canal.^12^ SmartLite Pro EndoActivator (SLP-EA, Dentsply Sirona) was developed as a second-generation sonic device, featuring polymer tips of variable cross-sections reaching up to 18,000 cycles per minute with elliptical motion to enhance irrigant agitation.^13^

Root canal sealers play a critical role in achieving a three-dimensional seal of the root canal system by improving adaptation to dentinal walls and flowing into anatomical irregularities.^14^ Beyond their contribution to overall sealing, penetration into dentinal tubules is considered a desirable feature, as it may help entomb or inactivate intratubular bacteria^15^ and promote micromechanical interaction with dentine, potentially enhancing filling retention.^16^

Confocal laser scanning microscopy (CLSM) is widely used to evaluate the depth and distribution of irrigant and sealer penetration into dentinal tubules.^17^ Although abundant evidence supports this method,^18,19,20^ most studies have overlooked the potential influence of dentine ageing on permeability and sealer penetration. The aim of the present study was to assess the effect of dentine ageing and two irrigant activation techniques, UA and SLP-EA, on the penetration of an endodontic sealer into dentinal tubules in the apical third. Therefore, the null hypothesis is that sealer penetration is not influenced by dentine ageing or by the activation/agitation technique employed (UA or SLP-EA).

## Methodology

This study was approved by the local research ethics committee (C.I 25/287-E).

### Specimen selection and preparation

For sample size calculation, a power of 0.90 and an α-type error of 0.05 were set to detect with a mean difference of 18.9% using Student’s t-test, based on data from a preliminary study, which estimated 20 specimens per group.

Eighty single-rooted freshly extracted teeth for orthodontic or periodontal reasons were obtained from donors aged 30–40 years (n = 40) and 60–70 years (n = 40). A buccolingual and a mesiodistal radiograph were obtained for each specimen to confirm the presence of a single canal and comparable internal anatomy. Teeth with endodontic treatment, root fractures, caries, resorption, immature apices, canal curvature >10°, or radiographically detectable canal irregularities, such as bifurcations or oval-shaped canals, were excluded. After cleaning and storage in 0.1% thymol for a maximum period of 2 months, the roots were standardised at 16 mm. The working length (WL) was defined as 1 mm short of the apical foramen and set at 15 mm. Subsequently, the external surface of the roots was coated with cyanoacrylate.

Canals were prepared with ProTaper Gold (Dentsply Sirona) to size F4 using the X-Smart Plus motor (Dentsply Sirona) following the manufacturer’s instructions. Between files, the canals were irrigated with 1 mL of 5.25% sodium hypochlorite (NaOCl, Dentaflux, Madrid, Spain) using a 30-G side-vented needle (DS Irrigation Needle, Dentsply Sirona) for 15 seconds at 1 mm from the WL.

### Irrigation activation protocols

Specimens from both age groups (30–40 and 60–70) were randomly assigned to two subgroups (n = 20) using computer software (http://www.random.org/) according to the activation/agitation method used during the final irrigation protocol.

- SLP-EA: 5 mL of 5.25% NaOCl were delivered 1 mm short of the WL and agitated for 1 min with the SLP-EA (Dentsply Sirona) using a medium tip (25/.04) at 18,000 cycles/min with vertical strokes 2 mm short of the WL. The procedure was repeated with 5 mL of 17% ethylenediaminetetraacetic acid (EDTA, CanalPro EDTA, Coltène Whaledent, Langenau, Germany).
- UA: 5 mL of 5.25% NaOCl were delivered 1 mm short of the WL and activated for 1 min with a #25/0.00 IrriSafe tip (Satelec/Acteon, Mérignac, France) attached to a P5 Newtron unit (Satelec/Acteon) at power setting 5, 2 mm short of the WL. The procedure was repeated using 5 mL of 17% EDTA.
- A final rinse with 5 mL NaOCl and 5 mL distilled water, without activation, was performed, and the canals were dried with F4 paper points (Dentsply Sirona).

### Obturation

The sealer BC Universal Sealer (FKG Dentaire, La Chaux-de-Fonds, Switzerland) was labelled with sodium fluorescein (Alquera, Madrid, Spain) at 0.01%. Obturation was performed using a single-cone technique: 0.05 mL of sealer was delivered with the manufacturer’s polymer tip into the middle third until visible coronally, and a Conform Fit F4 gutta-percha cone (Dentsply Sirona) was inserted to the WL. Coronal gutta-percha was compacted using the EQ-V plugger 50.04 (Meta Biomed, Cheongju, Korea) and a Buchanan #1 plugger (SybronEndo, Orange, USA).

Mesiodistal and buccolingual radiographs confirmed the obturation quality. The access cavity was sealed with composite (Ceram.x Spectra ST, Dentsply Sirona), and the specimens were incubated at 37°C and 100% humidity for 2 weeks.

### Sample processing and examination

Roots were sectioned 3 mm from the apex with a 0.2-mm diamond blade (Exakt Advanced Technologies) perpendicular to their axis to obtain slices 1.0 ± 0.1 mm thick. The coronal surface of each slice was polished using fine and superfine Super-Snap discs (Shofu Inc., Kyoto, Japan).

Root sections were imaged using an LSM 900 confocal laser scanning microscope (Carl Zeiss AG, Jena, Germany). Fluorescence was excited using a 488 nm laser line and detected within the 500–576 nm emission range. Images were acquired using either a low-magnification 5x objective (EC Plan-Neofluar 5×/0.16 M27) or a high-magnification 20x water-immersion objective (W N-Achroplan 20×/0.5 M27). All images were recorded at a resolution of 1024 × 1024 pixels, corresponding to a pixel size of 2.5 µm at 5x magnification and 0.38 µm at 20x magnification. Z-stacks were acquired, comprising approximately 12 optical sections with a step size of 4.5 µm. Images were recorded at an 8-bit depth using unidirectional scanning with line averaging set to 1x. The laser power, detector gain, and pinhole size were kept constant within each experimental set to ensure comparability across samples.

### Image analysis

Sealer penetration was quantified using Fiji (ImageJ 2.16.0; National Institutes of Health, Bethesda, USA) with a custom macro specifically developed for the quantitative assessment of sealer penetration. All analyses were performed by a blinded operator.

Confocal Z-stacks were imported using the Bio-Formats plugin and converted into maximum intensity projections, which were used for all subsequent analyses to capture the full extent of sealer penetration within each root slice (Figure 1).

**Figure 1:**
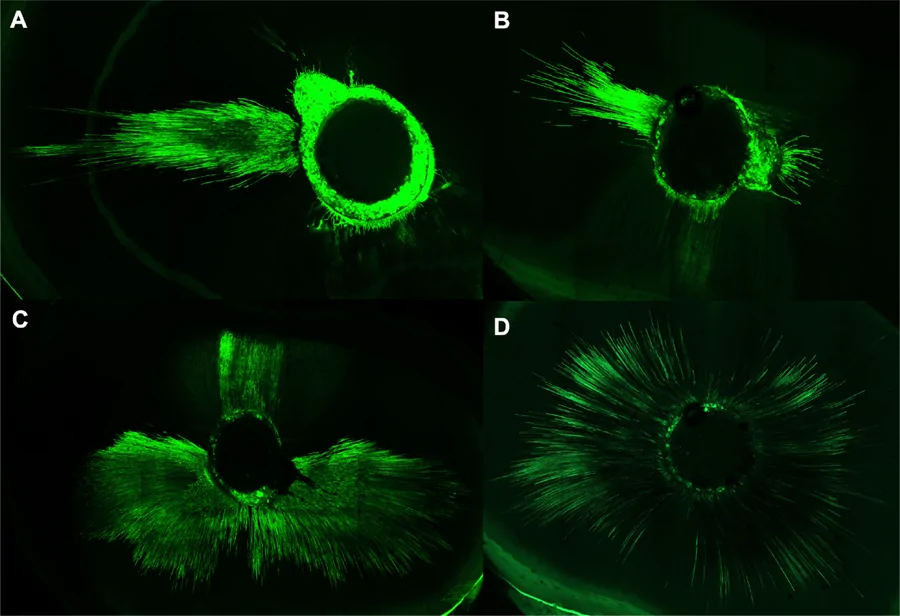
Representative images of each group.

(A) UA, 60–70 years. (B) SLP-EA, 60–70 years. (C) UA, 30–40 years. (D) SLP-EA, 30–40 years.

#### Sealer penetration analysis

High-magnification (20x) images were used for the quantitative assessment of sealer penetration into the dentinal tubules. The central canal region was manually excluded to restrict the analysis to the dentine tissue. Regions containing sealer penetration were interactively selected by the operator based on the presence of fluorescence signal. All subsequent processing steps were fully automated using macros.

Within each selected region, sealer fluorescence was segmented using the MinError automatic thresholding algorithm, generating binary masks corresponding to the infiltrated areas (displayed as red overlays; Figure 2C). The total penetration area (PA, µm^2^) was measured automatically and expressed as both an absolute value and a percentage relative to the total canal area measured in the corresponding low-magnification image.

**Figure 2:**
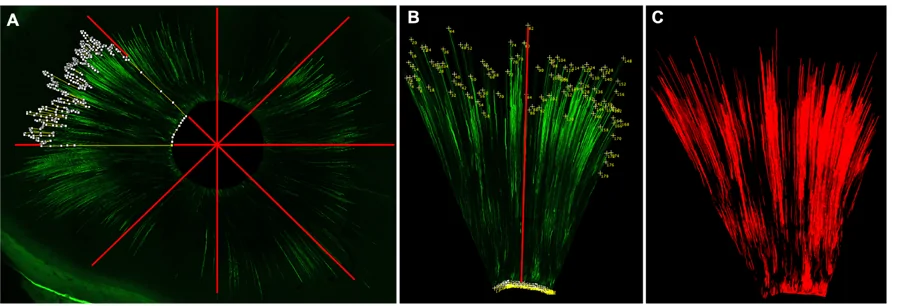
Quantification of sealer penetration.

(A) Sectoral mean penetration depth (S-MeanD). (B) Maximum penetration depth (MaxD, red line). (C) Total penetration area (PA, red overlay).

#### Penetration depth measurements

To standardise the depth measurements, each image was first aligned along the longitudinal axis of the canal. The orientation was guided by a single user-defined reference line drawn from the canal wall toward the root periphery. After rotation, penetration depth measurements were performed using a semi-automated workflow.

The macro divided the canal cross-section into eight equal angular sectors (45° each). Within each sector, penetration tracks were defined by pairs of user-selected points extending from the canal wall to the deepest detectable sealer fluorescence signal. Linear distances were automatically calculated by the macro.

Sectoral mean penetration depth (S-MeanD, µm) was calculated by averaging all penetration lengths within each sector, followed by averaging across the eight sectors (Figure 2A, B).

Maximum penetration depth (MaxD, µm) was defined as the longest individual penetration length measured within the slice (Figure 2B, red line).

#### Perimeter-based penetration analysis

To evaluate the circumferential distribution of sealer penetration, the canal perimeter was manually delineated using the Polygon tool, and then two additional concentric perimeter lines were drawn at fixed radial distances of 200 µm and 400 µm from the canal wall. Along each perimeter, fluorescent segments were identified and their cumulative lengths were quantified. The percentage of sealer penetration was calculated as the ratio between the total fluorescent segment length and the corresponding total perimeter length. This yielded penetration percentages at the canal wall (P%0), 200 µm (P%200), and 400 µm (P%400) depths (Figure 3).

**Figure 3:**
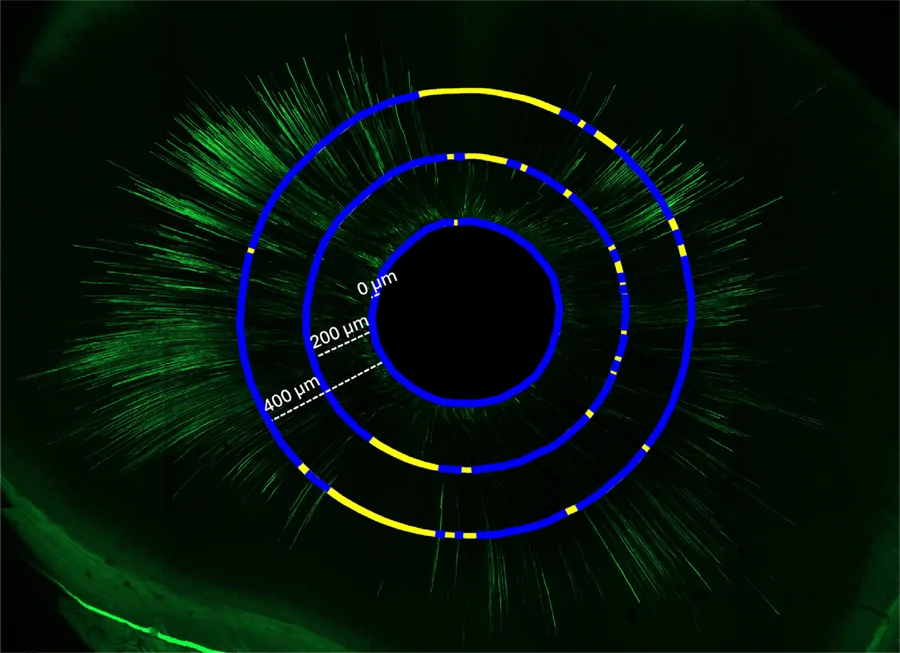
Percentage of sealer penetration along the canal perimeter. P%0 corresponds to the canal wall (0 µm), and P%200 and P%400 represent measurements at 200 µm and 400 µm within the dentine wall.

All quantitative measurements were automatically recorded in summary tables, and annotated overlay images illustrating penetration areas and depth measurements were generated and saved for quality control and visualisation.

## Statistical analysis

Data were analysed with SPSS 27.0 (IBM, New York, USA). Normal distribution was examined with the Shapiro–Wilk test. The distribution of PA, MaxD, S-MeanD, and P%400 in UA and SLP-EA groups was not compliant with normality nor was the distribution of PA, P%200, and P%400 in Age Groups. Therefore, a rank transformation was performed to enable the application of the parametric two-way ANOVA, to analyse the effect of Activation System, Age Group and their interaction on all penetration variables. A significance level of 0.05 was applied in all analyses.

## Results

### Effect of the activation system

No significant differences were detected between UA and SLP-EA in any of the penetration variables evaluated (Table 1). Both systems achieved comparable sealer penetration, with similar values for PA, MaxD and S-MeanD, and P%0, P%200 and P%400 (p > 0.05).

**Table 1:** Mean and standard deviation (SD) of penetration variables in groups defined by the two independent variables (activation systems and age groups) and results of the comparison with 2-way ANOVA (p value and effect size).

Total penetration area (PA) (µm^2^)																				
																			p	Effect size (eta_p_^2^)
30-40 y									60-70 y									Age Group	<0.001**	0.627
UA			SLP-EA			Total			UA			SLP-EA			Total			Activation System	0.877	0.000
N	Mean	(SD)	N	Mean	(SD)	N	Mean	(SD)	N	Mean	(SD)	N	Mean	(SD)	N	Mean	(SD)	Age Group* Activation System	0.176	0.024
20	2,278,586	(1,422,936)	20	1,815,812	(1,176,849)	40	2,047,199	(1309,984)	20	224,539	(180,548)	20	330,231	(287,801)	40	277,385	(243,101)			
**Maximum penetration depth (MaxD) (µm)**																				
																			**p**	**Effect size (eta_p_^2^)**
30-40 y									60-70 y									Age Group	<0.001**	0.541
UA			SLP-EA			Total			UA			SLP-EA			Total			Activation System	0.923	0.000
N	Mean	(SD)	N	Mean	(SD)	N	Mean	(SD)	N	Mean	(SD)	N	Mean	(SD)	N	Mean	(SD)	Age Group* Activation System	0.824	0.001
20	1,471	(383)	20	1,444	(471)	40	1,458	(424)	20	681	(325)	20	716	(291)	40	698	(305)			
**Sectorial mean penetration depth (S-MeanD) (µm)**																				
																			**p**	**Effect size (eta_p_^2^)**
30-40 y									60-70 y									Age Group	<0.001**	0.645
UA			SLP-EA			Total			UA			SLP-EA			Total			Activation System	0.342	0.012
N	Mean	(SD)	N	Mean	(SD)	N	Mean	(SD)	N	Mean	(SD)	N	Mean	(SD)	N	Mean	(SD)	Age Group* Activation System	0.230	0.019
20	747	(307)	20	728	(358)	40	738	(329)	20	155	(74)	20	210	(102)	40	183	(92)			
**Percentage of sealer penetration along the canal perimeter (P0) (%)**																				
																			**p**	**Effect size (eta_p_^2^)**
30-40 y									60-70 y									Age Group	<0.001**	0.202
UA			SLP-EA			Total			UA			SLP-EA			Total			Activation System	0.170	0.025
N	Mean	(SD)	N	Mean	(SD)	N	Mean	(SD)	N	Mean	(SD)	N	Mean	(SD)	N	Mean	(SD)	Age Group* Activation System	0.798	0.001
20	68.6	(14.6)	20	75.3	(15.0)	40	72.0	(15.0)	20	51.7	(20.1)	20	56.3	(22.4)	40	54.0	(21.2)			
**Percentage of sealer penetration along the perimeter at 200 µm (P200) (%)**																				
																			**p**	**Effect size (eta_p_^2^)**
30-40 y									60-70 y									Age Group	<0.001**	0.536
UA			SLP-EA			Total			UA			SLP-EA			Total			Activation System	0.045	0.052
N	Mean	(SD)	N	Mean	(SD)	N	Mean	(SD)	N	Mean	(SD)	N	Mean	(SD)	N	Mean	(SD)	Age Group* Activation System	0.834	0.001
20	49.7	(19.5)	20	58.2	(19.0)	40	54.0	(19.5)	20	14.8	(11.7)	20	22.7	(17.3)	40	18.7	(15.1)			
**Percentage of sealer penetration along the perimeter at 400 µm (P400) (%)**																				
																			**p**	**Effect size (eta_p_^2^)**
30-40 y									60-70 y									Age Group	<0.001**	0.579
UA			SLP-EA			Total			UA			SLP-EA			Total			Activation System	0.264	0,016
N	Mean	(SD)	N	Mean	(SD)	N	Mean	(SD)	N	Mean	(SD)	N	Mean	(SD)	N	Mean	(SD)	Age Group* Activation System	0.710	0.002
20	45.2	(21.1)	20	52.4	(21.1)	40	48.8	(21.2)	20	9.7	(10.2)	20	11.9	(13.2)	40	10.8	(11.7)			

** Indicates statistically significant difference between groups.

Activation system groups: UA (ultrasonic activation) and SLP-EA (SmartLite Pro EndoActivator); Age groups: 30-40 y and 60-70 y (years); N, sample size per group.

### Effect of age

In contrast, donor age influenced sealer penetration (Table 1). Younger teeth (30–40 years) showed significantly higher MaxD (1,458 µm vs 698 µm), S-MeanD (738 µm vs 183 µm) and PA than older teeth (60–70 years) (p < .001). Comparisons of P%0, P%200, and P%400 confirmed this trend (p < 0.001).

### Interaction of Activation System x Age Group

The interaction between the two variables did not have a statistically significant effect on any of the tested variables (p > 0.05) (Table).

## Discussion

The present study was designed to evaluate the influence of dentine ageing on tubular penetration of root canal sealer in the apical third of the root after using two different activation techniques (UA and SLP-EA). Significantly greater sealer penetration was observed in the 30–40 age group (738 μm of S-MeanD and 72% of P%0) than in the 60–70 age group (183 μm and 54% respectively), suggesting that dentine ageing affects tubular infiltration. This finding is consistent with previous investigations reporting an age-dependent reduction in dentine permeability using dye penetration models. Thaler et al. demonstrated that dye penetration areas significantly decreased with increasing age, particularly in the apical root sections, which they attributed to progressive dentinal sclerosis.^21^ More recently, Hahn et al. also reported significantly deeper dye penetration in younger teeth (< 30 years) than in older ones (> 60 years), especially in the buccolingual direction in mesial roots, suggesting that ageing alters the pattern of dentine permeability.^22^ This investigation evaluated this phenomenon using penetration of endodontic sealer rather than dyes. Given that sealers differ substantially from dyes in terms of particle size, viscosity, and flow characteristics, the present model reflects more closely the clinical conditions and provides a more relevant assessment of age-related changes in sealer–dentine interaction.

This age-related reduction in dentinal permeability has also been demonstrated for bacterial penetration.^7^ Although sealer penetration is lower in aged dentine, the reduced bacterial burden may mitigate its impact, resulting in comparable outcomes observed in older and younger teeth. This fact may explain why ageing does not compromise root canal treatment outcomes.^23^ Nevertheless, in vitro studies are more sensitive to structural variations, and therefore the different outcome observed in the present study should be taken into account for sample selection of experimental studies. Incorporating samples with uniform age could ensure more consistent results. It is worth noting that the use of an artificial ageing model, such as thermocycling, could facilitate the control of ageing-induced structural changes. This method, which is usually employed to predict the long-term behaviour of dental materials, may be an interesting approach for future research to evaluate the influence of dentinal changes.

Although donor’s age was the selected variable in the present study to investigate the influence of dentinal changes, other factors may also influence the development of dentinal sclerosis and dentine permeability. Local and functional conditions affecting the dentine–pulp complex may induce adaptive changes that promote progressive tubule occlusion and the formation of sclerotic dentine. Previous studies have shown that dentine permeability may vary among different tooth groups and can also be influenced by functional stresses experienced during mastication.^24^ Therefore, despite efforts to standardise the specimens, the potential effect of these additional factors should be considered when interpreting the present results.

Regarding activation systems, no significant differences were observed between sonic and ultrasonic activation. This is consistent with previous investigations that reported no differences between UA and EA at 3 mm ^19^ or 2 mm^20, 25^ from the apex. In contrast, Mancini et al. observed that EA promoted slightly greater smear layer removal than UA in the apical third.^26^ More recent studies evaluating SLP-EA showed antibacterial efficacy comparable to EA,^13^ whereas Huynh et al. reported that SLP-EA was less effective than laser-activated irrigation for debris removal.^11^ Although SLP-EA incorporates design improvements, our findings do not demonstrate a clear advantage over ultrasonic activation, underscoring the need for further studies. The absence of non-activated groups as negative controls could be acknowledged as a limitation of this study, since the inclusion of a control group would have provided a reference for comparison between techniques. Nevertheless, it is important to note that the advantage of activation over non-activation protocols in terms of sealer penetration has already been established in previous research^8, 19, 27^ and the objective of this study was to compare two specific activation techniques in two different age groups.

The analysis was restricted to the apical third, so findings cannot be extrapolated to middle or coronal regions, where dentinal structure and irrigant dynamics differ. Apical dentine has lower permeability, which limits both irrigant and sealer infiltration.^28,29^ This can be explained by dentinal sclerosis with ageing, characterised by progressive mineral deposition starting apically and extending coronally, leading to tubule obliteration.^6,30^ Since one of the objectives of this study was to evaluate the impact of dentine ageing, focusing specifically on the apical area, where more tubule obliteration is present, this seemed more relevant than the middle or coronal sections. Furthermore, the apical third was selected and prepared also with the aim of enhancing sample consistency. Instrumentation performed with relatively wide instruments is more likely to obtain a predictable round-shaped canal section in this region than at other levels of the root.^31^

Recent studies have raised concerns regarding the reliability of CLSM imaging for assessing sealer penetration due to the reported potential leaching of rhodamine B from the sealer even after setting, which could result in the diffusion of the fluorochrome into dentinal tubules or bind to water content, leading to an overestimation of penetration depths and false-positive signals.^18, 32^ Considering these factors, sodium fluorescein was selected, given its validated use in CLSM-based assessments as it reduces the likelihood of dye-related artifacts.^33, 34, 35^ This dye exhibits favourable fluorescence intensity and optical properties and, when properly incorporated, enables reliable visualisation of sealer or irrigant penetration without the diffusion-related artifacts associated with rhodamine B. A low concentration (0.01%), below the 0.1% commonly employed,^19,25,33,36,37^ allowed adequate fluorescence while reducing the potential effect on the material’s properties.

The most appropriate method to evaluate sealer penetration is still questioned. MaxD alone may not be clinically relevant, since localised deep penetration does not guarantee effective sealing.^38^ Previous publications have evaluated penetration by measuring specific points along the canal wall, reporting the exact depth reached at each location.^25,36,37^ Instead of using point-based measurements, the methodology employed in this study divided the canal into eight sectors and quantified all tubules within each region (S-MeanD). This approach allows a more representative quantification of sealer distribution, since it accounts for all penetration events within a given area and differentiates mesiodistal from buccolingual surfaces. This distinction is important as sealer penetration tends to be lower mesiodistally due to the so-called butterfly effect, characterised by progressive dentinal sclerosis in this direction.^6^

Other parameters, such as percentage of sealer penetration, provide more meaningful information.^39^ In the present study, the percentage of sealer penetration at different dentine depths was considered the primary outcome, as it provides information not only about the extent of penetration but also about its circumferential pattern within the dentinal wall. To further evaluate distribution, perimeter analysis was performed at different depths (P%200 and P%400), around the canal wall (P%0), as described by Keles et al.,^40^ providing complementary information on radial distribution. This analysis captures not only depth but also how homogeneously it is distributed along the canal wall, offering a comprehensive representation of sealer behaviour within dentinal tubules.

Another strength of this study is the use of z-stacks, which provide a more comprehensive visualisation of sealer distribution across different depths. CLSM enables three-dimensional reconstruction by combining multiple optical sections,^17^ in line with multilayer imaging approaches previously applied in several sealer penetration studies.^36,41^ This procedure offers a more accurate and comprehensive understanding of the extent and variability of penetration throughout the sample. Penetration patterns varied significantly across different depths within the same sample, as shown in Figure 4, underscoring the potential limitations of a single-plane analysis.

**Figure 4:**
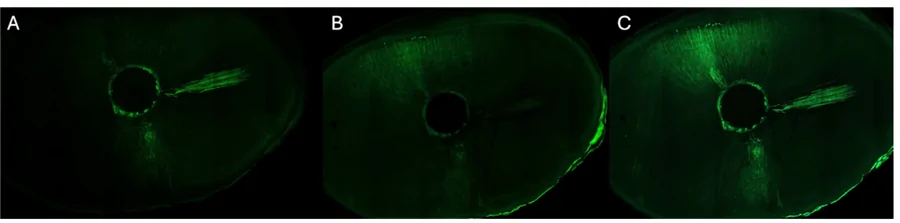
Representative images from the acquired confocal stack. Sequential optical sections captured at different focal depths.

(A) Image of a section at 10 µm from the surface. (B) Image of a section located 27 µm deeper than Image A. (C) Maximum intensity projection combining all acquired sections in the stack.

## Conclusion

Within the limitations of this study, it may be concluded that dentine ageing affects the penetration of endodontic sealer into dentinal tubules in the apical third of the root canal, regardless of the technique used to activate the irrigant (UA or SLP-EA).

## Data Availability

The datasets generated during and/or analyzed during the current study are available from the corresponding author on reasonable request.
